# Hypersensitivity of Vestibular System to Sound and Pseudoconductive Hearing Loss in Deaf Patients

**DOI:** 10.1155/2014/817123

**Published:** 2014-03-03

**Authors:** Seyede Faranak Emami

**Affiliations:** Department of Audiology, Faculty of Rehabilitation, Hamadan University of Medical Sciences and Health Services, Hamadan 16657-696, Iran

## Abstract

The objective of this cross-sectional study is to compare bone-conducted low-frequency hearing thresholds (BClf) to cervical vestibular evoked myogenic potentials (cVEMPs) findings in prelingual adult deaf patients. The fifty participants (100 ears) included twenty healthy controls and thirty other subjects selected from patients who presented with bilateral prelingual deafness to Department of Audiology of Hamadan University of Medical Sciences and Health Services (Hamadan, Iran). Assessments comprised of audiological evaluations, cVEMPs, and computerized tomography scans. Twenty deaf patients (*forty affected ears*) with bilateral decreased vestibular excitability as detected by abnormal cVEMPs revealed that BClf hearing thresholds were completely absent. Ten deaf patients (*twenty unaffected ears*) with normal cVEMPs reported a sensation of the sound at BClf hearing thresholds (the mean for 250 Hz*=*41 dBHL and for 500 Hz*=*57.75 dBHL). Multiple comparisons of mean p 13 latencies, mean n23 latencies and peak-to-peak amplitudes between three groups were significant (*P* = 0.01 for all, one-way ANOVA test). Multiple Comparisons of mean BClf between three groups were significant (*P* = 0.00, One-way ANOVA test). *Conclusion*. Hypersensitivity of vestibular system to sound augments BClf hearing thresholds in deaf patients. The sensation of the sound at low frequencies may be present in patients with total deafness and normal vestibular function (predominantly saccule). This improvement disappears when saccular function is lost.

## 1. Introduction

The mammalian inner ear contains sense organs responsible for detecting sound, gravity, and acceleration. Of these organs, the cochlea is involved in hearing, while the otolith organs (saccule and utricle) serve to detect linear acceleration [1]. Recent evidences from human show that the saccule has acoustic sensitivity to sound [2–4], which can contribute to the affective quality of loud low frequencies [4]. Saccular stimulation to air-conducted sound has a compensatory role for cochlear hearing in noisy conditions [3]. Saccule not only responds best to low frequency high-intensity air-conducted sound, but also, in clamor conditions, may contribute to the hearing of this frequency band [2]. Saccular hearing is an effective reinforcer for cochlear hearing [4]. It can cooperate to frequency and intensity discrimination [5, 6].

The otolith organs have a mechanical tuning due to their elastic and inertial properties and the band-width of their mechanical response extending to 500 Hz. The sensitivity of the human vestibular system to bone-conducted stimulation exceeds that of the cochlea for low frequencies, which is a particular means of activating the vestibular system in normal subjects [7]. Hair-cells are known to exhibit electrical resonance in low-frequency range due to the interaction of transduction and basolateral currents [8].

Saccular sensitivity to air and bone-conducted stimulation is revealable by cVEMPs [7]. It remains intact in humans who have discrete genetic pathologies of the cochlea and semicircular canals and can still be evoked from patients, if they have a preserved otolithic organ [8]. Then, the aim of the present study is to compare bone-conducted low-frequency hearing thresholds to cVEMPs findings in Hamadanian prelingual adult deaf patients.

## 2. Materials and Methods

This cross-sectional study involved twenty healthy subjects compared to thirty bilateral congenital deaf cases (on historied medical and audiological evaluations, and educational backgrounds in deaf schools), which were volunteers who presented to the audiology department of Hamadan university of medical sciences and health services (Hamadan, Iran), from December 2012 to April 2013, and the study was approved by the Hamadan university ethics committee.

The exclusion criteria were history of ear infections and middle ear diseases, dizziness or vertigo, which could interfere with cVEMPs measurements, and the possibility of semicircular canals dehiscence on imaging of the temporal bone (it is a clinical entity of unexplained conductive hearing loss in the presence of both normal middle-ear compliance and stapedial-reflex thresholds, the patients complain of vertigo induced by loud sound and/or changes in middle-ear pressure [7, 9]).

The inclusion criteria were bilateral prelingual deafness, normal middle ear pressure, lack of vertigo induced by loud sound, and nonexistence of vertigo induced by changing of middle-ear pressure.

Our criteria for normal vestibular sensitivity to sound were normal air-conducted cVEMPs. It can be possible to separate superior semicircular canal dehiscence from healthy persons. In this form, the amplitudes are larger than normal persons [9, 10]. But, bone-conducted cVEMPs is less useful diagnostically in superior semicircular canal dehiscence and has lesser abnormalities [5].

## 3. Assessments

A total of one hundred ears were evaluated; testing was performed bilaterally. The recording procedures consisted of otoscopic examination, pure tone audiometry, tympanometry, cVEMPs, and computerized tomography scans. All participants were asked to read and sign a consent form in order to conform with the local ethical committee guidelines of Hamadan university of medical sciences and health services. The tests were performed on the same day; in each step of the evaluation, when the procedure was completed for the one test, subjects were given a short break and the whole procedure was repeated for another. The devices comprised of diagnostic pure tone audiometry (Inventis; Harp plus), impedance acousticmetry (Maico MI 34), full system of auditory-vestibular evoked potentials (Labat Epic-plus).

The computerized tomography scans made to rule out the probability of the semicircular canals dehiscence syndrome. *Audiologic tests* were performed by audiology staff of our place on the same day. In every morning and at first stage of each evaluation, we were controlled the calibration of audiologic systems. The staff did not know about the case or control subjects (heading of our research was blind), and testing was randomized. Before starting of the assessment, we ensured that all tympanic membranes were intact. During the process, we evaluated the test results, and made sure that testing was done properly. If there was a problem excluding the possibility of false responses, the patients underwent several retesting (by an expert clinician).

The middle-ear pressures were obtained between the limits of ±50 dapa [10]. Also, the assessment of vertigo induced by changing of middle-ear pressure was done. The cVEMPs results for the normal group were used as normative data (mean ± two standard deviations). The latencies longer than the calculated upper limit were interpreted as abnormal. Absence of a meaningful waveform with p13 and n23 was also considered as an abnormal finding [7].

Pure tone audiometric thresholds obtained (for air-conducted sounds) from all subjects over the frequency range of 250, 500, 750, 1000, 2000, 4000, and 8000 Hz, and 250 to 4000 Hz (for bone-conducted vibrations), respectively [11]. The upper limits of the bone-testing at each frequency for the calibrated transducer consisted of

(1) $$\begin{array}{c} 250\;\text{Hz}=45\;\text{dBHL},\;\;500\;\text{Hz}=65\;\text{dBHL},\\ 750\;\text{Hz}=70\;\text{dBHL},\;\;1000\;\text{Hz}=75\;\text{dBHL},\\ 2000\;\text{Hz}=80\;\text{dBHL},\;\;4000\;\text{Hz}=75\;\text{dBHL}. \end{array}$$

The possibility of vibrotactile responses for bone-conducted low-frequencies was ruled out during audiometric testing with using of insert (ER-3A) earphones [11]. In addition, we gave instructions to patients about bone-conducted hearing versus feeling of vibrotactile stimuli. Since, the stimulus energy may seep around the earphone cushion and travel via air to the other ear. So, we used of ER-3A insert earphones, which increased interaural attention of acoustic signals and the need for contralateral masking was eliminated at 250, 500_HZ_. The absence of vertigo induced by loud sound was evaluated. The upper limits of the air-testing at each frequency for the calibrated insert (ER-3A) earphones consisted of

(2) $$\begin{array}{c} 250\;\text{Hz}=100\;\text{dBHL},\;\;500\;\text{Hz}=110\;\text{dBHL},\\ 750\;\text{Hz}=120\;\text{dBHL},\\ 1000\;\text{Hz}=120\;\text{dBHL},\;\;2000\;\text{Hz}=120\;\text{dBHL},\\ 4000\;\text{Hz}=120\;\text{dBHL},\;\;8000\;\text{Hz}=100\;\text{dBHL}. \end{array}$$

## 4. Data Analyses

All analysis was done by means of the statistics software SPSS_17_. Kolmogorov-Smirnov test was used for evaluation of normal test distribution. One-way ANOVA was used to compare findings among the three groups. Tukey's least significant difference (Tukey HSD) test was chosen as the post hoc test. *P* value of < 0.05 was considered to indicate statistical significance.

## 5. Results

We evaluated twenty healthy subjects including 10 females and 10 males (20–39 years old, mean age of 24 years). Deaf patients consisted of thirty cases: 18 females and 12 males with bilaterally congenital deafness (23–39 years old, mean age of 28 years). All of the patients and subjects had no head and neck exam, with normal otoscopic exam. Thin-sliced CT scans of the temporal bone revealed normal middle and inner ear anatomy. No radiological sign of thin tegmen, dehiscence of any of the semicircular canals, or large vestibular aqueduct was noticed.

Tympanometry revealed normal pressure and volume in all the tested ears. No sign of pressure- or sound-induced nystagmus or vertigo. All of the tested ears had negative fistula test and/or Tullio phenomenon.

**Figure 1:**
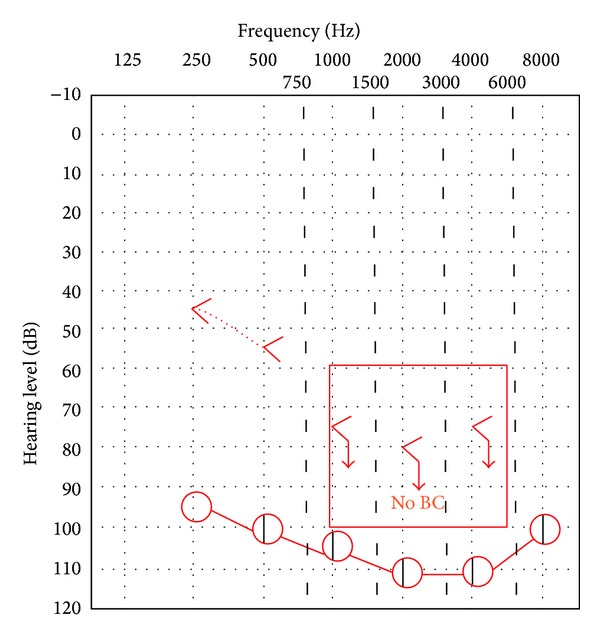
Bone-conducted low frequency hearing thresholds and pseudoconductive hearing loss in a deaf patient. Bottom trace: air-conducted hearing thresholds, top trace: bone-conducted hearing thresholds.

The normal subjects had normal pure tone audiograms, and normal cVEMPs values (Table 1). The deaf patients (60 ears) had bilateral deafness (air-conducted hearing thresholds were more than 90 Dbhl  [11]). In twenty deaf patients with bilateral abnormal cVEMPs values (40 affected ears), bone-conducted hearing thresholds as a sensation of the sound were bilaterally *lost*. The other deaf patients (ten cases = 20 unaffected ears) had normal cVEMPs, with presence of hearing sensitivity at BClf hearing thresholds (mean for 250 Hz = 41 dBHL, minimum = 35 dBHL, maximum = 45 dBHL, and mean for 500 Hz = 57.75 dBHL, minimum = 55 dBHL and maximum = 65 dBHL) (Table 2). Some of them, whose air-conduction hearing thresholds were 100 dBHL at frequency of 250 Hz, gave responses to BClfstimuli at the maximum output level of 45 dBHL. This hearing sensation to bone-conducted stimulations induced the discrepancy between air- and bone-conducted hearing thresholds (Figure 1), which disappeared over frequencies higher than 500 Hz. The whole deaf patients had not sound and/or pressure evoked vertigo, disequilibrium, and vestibuloocular reflexes in response to sound.

## 6. The Main Outcome Measures

Multiple comparisons of mean p13 latencies, mean n23 latencies, and mean peak-to-peak amplitudes of the cVEMPs between three groups (affected, unaffected, and normal ears) were significant (*P* = 0.01 for all, one-way ANOVA test).

Comparisons of mean p13 latencies (*P* = 0.02, Tukey HSD), mean n23 latencies (*P* = 0.03, Tukey HSD), and mean peak-to-peak amplitudes (*P* = 0.04, Tukey HSD) in affected versus unaffected and normal ears were significant.

Multiple comparisons of mean BClf  between three groups were significant (*P* = 0.00, One-way ANOVA test). Comparisons of mean BClf in affected versus unaffected and normal ears were significant (*P* = 0.05, Tukey HSD). There were no significant differences in age and sex between three groups (*P* > 0.05 for all, one-way ANOVA test).

## 7. The Main Results

Hypersensitivity of vestibular system to sound augments BClf hearing thresholds in deaf patients. The sensation of the sound at low frequencies may be present in patients with total deafness and normal vestibular function (predominantly saccule). This improvementdisappears when saccular function is lost.

## 8. Discussion

In this paper, I reported that forty* affected ears* of the deaf patients with decreased vestibular excitability as detected by abnormal cervical vestibular evoked myogenic potentials (cVEMPs) had bone-conducted low-frequency (BClf) hearing thresholds were completely absent, whereas, twenty unaffected ears of them with normal cVEMPs findings reported the presence of a sensation to sound at BClf hearing thresholds. I concluded that the auditory sensitivity of the saccule augments BClf hearing thresholds in deaf patients.

The pattern of hypersensitivity to sound stimulation is consistent with the clinical sign known as Tullio phenomenon (the generation of vestibular symptoms during exposure to high intensity sounds). The cause is usually a fistula in the middle or inner ear, allowing abnormal sound-synchronized pressure changes in the balance organs [7]. Tullio phenomenon is also one of the common symptoms of superior canal dehiscence syndrome and invariably induces a characteristic conductive hyperacusis, that is, better-than-normal hearing thresholds for bone conduction in combination with a clear air-bone gap (up to 60 dB) in patients with sensory hearing loss, with normal acoustic reflexes and word recognition performance [9].

Afferent vestibular fibers in primates and humans can be activated by either sound or vibration [8]. The perception of bone-conducted sounds is mediated through the vestibular endings in the otolith organ [7]. However, bone vibration at a given perceptual intensity is a more effective vestibular stimulus than air-conducted sound [12]. This sensation is explainable by BClf hearing thresholds [13]. Bone-conducted vibration of the head causes linear acceleration stimulation of both inner ears and this linear acceleration is an effective way of selectively activating otolithic afferent neurons [14]. The response of the skull to vibration is complex and, while the direction of fluid movement through the cochlea is constant, transmission of vibration to the vestibular end-organs is likely to depend on the direction and frequency of the applied stimulus [13].

On the other hand, the inertia of the middle ear is not an important contribution to the perception of bone-conducted sound for frequencies below 1.5 kHz. The fluid flow at the round window, rather than at the oval window, reflects the stimulation of the basilar membrane with bone conduction stimulation [15]. However, all the vestibular end-organs (three canals and two maculae) responded to sound [16]. Among the five end-organs, the saccular macula showed the lowest thresholds [13]. The best frequencies did not exceed 1000 Hz to sound and 500 Hz to vibration [16]. On the other hand, AC-cVEMPs can be recorded at 80–95dBnHL, but cVEMPs can be elicited at lower sound levels (70dBnHL) for stimuli delivered by bone conduction [12]. Also, the difference between hearing thresholds in pure tone audiometry and cVEMPs thresholds remains much larger for AC-sounds than for BC-vibrations [7].

The saccule may be the most sound-sensitive among the vestibular end-organs. Indeed, Single neuron studies in animals have shown that semicircular canal neurons are rarely activated by levels of bone-conducted vibration at low frequencies, which generate vigorous firing in otolithic irregular neurons. Also, bone-conducted sound-sensitive afferents can be of utricular origin, because many of the bone-conducted sound-sensitive afferents are in the superior vestibular nerve, and they are sensitive to roll tilts, [16]. Hence, the vestibular and cochlear sensory organs have similar mechanical receptors and transducers but their specific ranges of sensitivity lie widely apart and tuning seems to be a special cochlear property.

After all, I believe that in the deaf the saccular stimulation to sound plays an auditory role. The auditory sensitivity of the saccule augments BClf hearing thresholds in deaf patients. BClf sounds may be mediated through vestibular endings in the saccule. In this regard, many perceptive audiograms with a rapidly sloping curve display bone thresholds which are exceptionally good up to 500 Hz and definitely better than the corresponding air thresholds.

This improvement for BClf disappears over frequencies higher than 500 Hz. The striking discrepancy between air and bone thresholds over the lower frequencies is generally present in cases where vestibular excitability is within normal limits. Then, sensation of the sound at low frequencies may be present in patients with total deafness and normal vestibular function.

## 9. Implications for Clinical Practice

The profoundly deaf subjects with a normally functioning vestibular system may obtain useful information from BClf sounds when stimulated adequately. It can be possible for a totally deaf person to process acoustic stimulation via a BClf saccular implant and, with training, learn to adapt to and use any vestibular information to further distinguish the acoustic signal.

## Figures and Tables

**Table 1:** The mean latency values and the mean peak-to-peak amplitudes in three study groups.

Group	Number	Latency of p13 (ms)	Latency of n23 (ms)	Peak-to-peak amplitude (*μ*v)
Normal ears	40	12.9 ± 1.4	22.2 ± 1.6	67.9 ± 9.8
Affected ears of deaf patients	40	19.5 ± 1.5	28.5 ± 1.2	10.1 ± 6.7
Unaffected ears of deaf patients	20	13.1 ± 0.95	21.5 ± 1.1	68.6 ± 6.3

**Table 2:** Air- and bone-conducted hearing thresholds (250 Hz and 500 Hz) in unaffected ears.

Case number	ACHT (dBHL)		BCHT (dBHL)	
	250 Hz	500 Hz	250 Hz	500 Hz
1	95	95	40	65
2	90	95	35	55
3	100	105	45	55
4	100	110	40	65
5	95	110	45	65
6	90	110	35	50
7	100	105	45	60
8	100	115	45	65
9	95	95	40	50
10	95	95	45	60
11	100	100	45	50
12	95	105	40	60
13	100	110	40	55
14	95	115	35	55
15	90	100	35	40
16	100	110	45	60
17	100	120	45	65
18	95	120	45	65
19	100	100	40	60
20	95	105	35	55

The mean of air-conducted hearing thresholds (ACHT): 250 Hz = 96.5 dBHL, 500 Hz = 106 dBHL.

The mean of bone-conducted hearing thresholds (BCHT): 250 Hz = 41 dBHL, 500 Hz = 57.75 dBHL.

## Abbreviations

cVEMPs: Cervical vestibular evoked myogenic potentials
BClf: Bone-conducted low frequency.
